# Understanding Tail-Biting in Pigs through Social Network Analysis

**DOI:** 10.3390/ani8010013

**Published:** 2018-01-15

**Authors:** Yuzhi Li, Haifeng Zhang, Lee J. Johnston, Wayne Martin

**Affiliations:** 1 West Central Research and Outreach Center, University of Minnesota, Morris, MN 56267, USA; zhanghaifenga10@126.com (H.Z.); johnstlj@umn.edu (L.J.J.); 2 Division of Agriculture, University of Minnesota Extension, St. Paul, MN 55108, USA; marti067@umn.edu

**Keywords:** social network analysis, tail-biting, pigs

## Abstract

**Simple Summary:**

Pigs are social animals that form social structures to maintain group stability. Grouping pigs without consideration of their social preferences may result in the development of abnormal behaviors such as tail-biting. This study was conducted to evaluate the association between social structure and incidence of tail-biting in pigs. Pigs were grouped according to their relatedness: (1) all pigs in a pen were related (born and raised by the same mother pig and called littermates); (2) all pigs in a pen were not related (born and raised by different mother pigs—non-littermates); and (3) half of the pigs in a pen were related (all pigs in a pen were born and raised by two mother pigs with equal number of pigs from each mother—half-group of littermates). Results indicate that littermates were less socially connected among themselves within a pen by spending less time lying together with their pen-mates than non-littermates. Littermates had a higher incidence of tail-biting compared to non-littermates. These results suggest that less social connection with pen-mates might predispose littermate pigs to the development of tail-biting. This pilot study demonstrates that social network analysis may provide us new insights into development of tail-biting.

**Abstract:**

The objective of this study was to investigate the association between social structure and incidence of tail-biting in pigs. Pigs (*n* = 144, initial weight = 7.2 ± 1.57 kg, 4 weeks of age) were grouped based on their litter origin: littermates, non-littermates, and half-group of littermates. Six pens (8 pigs/pen) of each litter origin were studied for 6 weeks. Incidence of tail injury and growth performance were monitored. Behavior of pigs was video recorded for 6 h at 6 and 8 weeks of age. Video recordings were scanned at 10 min intervals to register pigs that were lying together (1) or not (0) in binary matrices. Half weight association index was used for social network construction. Social network analysis was performed using the UCINET software. Littermates had lower network density (0.119 vs. 0.174; *p* < 0.05), more absent social ties (20 vs. 12; *p* < 0.05), and fewer weak social ties (6 vs. 14, *p* < 0.05) than non-littermates, indicating that littermates might be less socially connected. Fifteen percent of littermates were identified as victimized pigs by tail-biting, and no victimized pigs were observed in other treatment groups. These results suggest that littermates might be less socially connected among themselves which may predispose them to development of tail-biting.

## 1. Introduction

Animal agriculture has been shaped by the general public’s concerns about animal welfare. Production procedures that cause pain in animals are coming under scrutiny. One such procedure is tail-docking in pigs. The European Union Council Directive 2008/120/EC [1] recommended but did not mandate the prohibition of tail-docking on a routine basis. As a result, some member countries of the European Union have implemented stricter rules on tail-docking than recommended in the European Union Council directive [2]. In the United States, most pigs are tail docked, which is a concern for consumers and the general public. In fact, some market supply chains in the U. S. have prohibited tail-docking of pigs, such as the All-Natural Pork produced for Niman Ranch [3] and certified organic pork produced for Organic Valley/Prairie [4].

While tail-docking greatly reduces the incidence of tail-biting, it does not eliminate the problem. Research results and on-farm observations [5,6] have suggested that tail-biting occurs more commonly in conventional production systems where pigs are housed indoors on fully slatted floors, rather than in alternative production systems where pigs are kept on bedded floors or outdoors. In fact, tail-biting also occurs among pigs raised with access to outdoors [7] suggesting that indoor housing, high stocking density, and lack of bedding in conventional systems are not the root causes of tail-biting. Although many researchers have committed significant effort to identify contributing factors and seek solutions, the problem of tail-biting remains [8,9]. In fact, tail-biting is caused by multiple factors, including genetics, nutrition, environment, and management [10,11]. The challenge is that tail-biting occurs sporadically. Many times, pigs in some pens may have suffered severe tail-biting while pigs in other pens within the same barn did not have tail-biting problems at all. One aspect of the tail-biting problem that has been overlooked in past research is the social dynamics of pigs in a group. Pigs are social animals that form social structures to maintain group stability. However, in commercial production, pigs are grouped without much consideration of their social preferences and social rules. Conceivably, mismatch of pigs in a group may result in unbalanced social structure, which induces abnormal behaviors such as tail-biting. In this pilot study, we explored the association between tail-biting and social structure by using Social Network Analysis.

Social Network Analysis (SNA) is a method of studying social structures at the group level and social positions at the individual level [12]. In other words, SNA tells us who is connected to whom in a group and by what relationship. A particular strength of SNA is that it offers the ability to examine and quantify social structures and positions through interactions at the individual level [13]. Quantitative measures obtained from SNA can be used to describe and compare social structures, to identify factors that change social structure, and to examine the effect of social structure on the behavior of individuals [14,15,16]. For social animals, including swine, the behavior of individuals affects and is affected by the behavior of others within their social network. Social interactions can spread (or transmit) certain behaviors within a group through learning. However, social interactions may also create new behaviors, sometimes abnormal behaviors, as the animal adopts to or copes with stressful conditions. Although SNA is a powerful tool to reveal social structure and the roles of individuals within the group, it has not been applied widely to solve animal welfare problems [17,18]. The objective of this pilot study was to identify association between occurrence of tail-biting and social structures in pigs. To our knowledge, this study was the first to use SNA in an investigation of tail-biting in pigs. This novel approach might provide us new insights into development of tail-biting that traditional approaches have been unable to uncover.

## 2. Materials and Methods

This study was conducted at the University of Minnesota’s West Central Research and Outreach Center in Morris, Minnesota. The Institutional Animal Care and Use Committee of the University of Minnesota reviewed and approved the experimental protocol of this study (IACUC# 1602-33462A).

### 2.1. Animals, Housing and Management

Pigs (Landrace × Yorkshire × Duroc) were born to 30 sows during a one-week period on the research farm in a confinement farrowing barn. The farrowing barn had two mirror-image rooms, each having 16 individual farrowing crates with creep areas on plastic-coated wire floors. Within 24 h after birth, all piglets were weighed, had needle-teeth clipped, were injected with iron, and male piglets were castrated without receiving any anesthetics or analgesics. At the same time, cross-fostering was conducted to avoid litter size greater than 14 piglets. Tail-docking was not performed so that all piglets had their tails intact. Room temperature in the farrowing barn was maintained by a ventilation and heating system at 20 ± 1 °C and piglets were provided with supplemental heat by electric heat lamps. There was one nipple drinker in each pen for piglets, but no creep feed was provided during the lactation period. Piglets were weaned at about 4 weeks of age and transferred to a confinement nursery barn. The nursery barn had two mirror-image rooms, with 32 pens in each room. Each nursery pen was equipped with a 5-space dry feeder and one bowl drinker. The nursery pen housed 8 pigs/pen on plastic slotted floors and provided floor space allowance of 0.34 m^2^/pig excluding the space occupied by the feeder. Room temperature in the nursery barn was maintained at 28 ± 1 °C when pigs entered the barn and decreased to 23 ± 1 °C over the subsequent 6-week period. All pigs had free access to fresh water and corn-soybean meal diets fed in mash form formulated according to NRC recommendations [19]. Light period was 8 h daily starting at about 0800 h in both the farrowing and the nursery barn. No environmental enrichment was provided in the farrowing barn or the nursery barn.

### 2.2. Experimental Treatments

Littermates may form strong associations during the lactation period, which may affect their social structures in later phases of production. To create different social structures, pigs were grouped according to their litter origin.

Three treatment groups were formed based on litter origin at weaning:

1. Littermates: All 8 pigs (4 barrows and 4 gilts) in a nursery pen were farrowed and nursed by the same sow. Littermates were not mixed with unfamiliar pigs when entering the nursery barn.
2. Half-group of littermates: Eight pigs (4 barrows and 4 gilts) in a pen were farrowed and nursed by two sows with equal number of pigs from each sow. Four pigs (2 barrows and 2 gilts) from each of two litters were mixed when entering the nursery barn.
3. Non-littermates: Eight pigs (4 barrows and 4 gilts) in a pen were farrowed and nursed by eight different sows, and were mixed when entering the nursery barn.

The study started when the pigs were allocated into nursery pens after weaning at 4 weeks of age. Pigs (*n* = 144, initial weight = 7.2 ± SD 1.57 kg) were selected at weaning according to their litter origin, tail injury, sex, and body weight for the study. There were 6 pens for each treatment group and 8 pigs/pen with equal number of barrows and gilts. All pigs had no tail damage upon entering the study. An effort was made to achieve similar average weight and in-pen weight variation (coefficient of variation) across all pens. Once assigned, pigs were housed with their pen-mates for 6 weeks until the end of the study at 10 weeks of age.

### 2.3. Data Collection

#### 2.3.1. Growth Performance

Pigs were weighed individually at weaning (4 weeks of age), 5 weeks, 7 weeks, and at the conclusion (10 weeks of age) of the study. Feed additions to each pen were weighed and recorded. Excess feed in the feeder was weighed at the time of weighing pigs. Average daily gain was calculated on an individual pig basis, and average daily feed disappearance and gain-to-feed ratio were calculated on a pen basis for the entire nursery period.

#### 2.3.2. Tail Scores

Pigs were assessed for tail injury at the initiation of the study, and once a week thereafter. Tail injury was assessed using a modified 0 to 4 scoring system based on the method of Kritas and Morrison [20]: score 0 = no damage; score 1 = healed lesions (scars or small scabs); score 2 = evidence of chewing or puncture wounds with visible blood but no signs of infection; score 3 = evidence of chewing or puncture wounds with visible blood and signs of infection, or partial loss of the tail without signs of infection; score 4 = partial or total loss of the tail with signs of infection. In addition to the weekly assessment of tail injury, all pigs were observed once daily throughout the study for routine health checks. Once any pig with visible blood on a tail was observed, all pigs in that pen (referred to as ‘tail-biting pen’) were assessed for tail injury once daily until all pigs in the pen were scored 0 or 1. Pigs with a tail score of 2 or higher at any time point during the nursery period were defined as victimized pigs. In this study, the first victimized pig was observed at week 9 of age. During the entire period of data collection, pigs in tail-biting pens were scored daily for 2 to 3 days after the first victimized pig in the pen was observed.

#### 2.3.3. Behavior

To construct social networks of pigs within pens, behaviors of pigs were video recorded using Infrared Color Bullet Cameras (TruVision High Definition Camera—1080p Bullet, United Technologies, Farmington, CT, USA) at 12 frames/s. Each camera was affixed to the top of a side wall with an angle downward to cover the entire area of two adjacent pens. All cameras were connected to a computer equipped with a time-lapse DVR device and video recording software (Geo Vision Multicam Digital Surveillance System V8.2; USA Vision Systems Inc., Irvine, CA, USA). Video recording took place twice during the nursery period. The first video recording took place when pigs were 6-weeks old, 2 weeks after entering the nursery barn to allow pigs to overcome weaning stress, adapt to the new environment, and establish stable social structures [21]. A week after the first video recording, pigs within a pen were moved to another identical pen within the same room. Moving pigs to new pens was designed to evaluate whether pigs were associated with each other due to their social preference or due to their preference for a particular location within their current pen [22]. The second video recording was conducted at 8 weeks of age, a week after the pigs were moved to their new pen. Each video recording started at about 0900 h and lasted 6 h. Before filming, each pig was marked uniquely to allow identification of individual pigs during video recording.

The video recordings were viewed by a trained researcher to determine association interactions among pigs. To avoid subjective bias on data collection, the researcher was blind to treatment groups when viewing the video recordings. Pigs were considered associated with each other if they were lying together frequently [22]. Lying together was defined as two pigs lying with 50% or more of their bodies in physical contact with each other or the head of a pig was in contact with another pig. The video recordings were scanned at 10 min intervals using the instantaneous scan sampling method described by Martin and Bateson [23]. At each scan, identification of pigs, postures of pigs (lying = 1; not lying = 0), and lying together (yes = 1; no = 0) were registered. Frequency of lying events was used to estimate the time budget for the lying posture using the following equation: lying time budget (%) = the number of times that a pig was observed in the lying posture/the total number of observations (scans) × 100% [23]. Data of lying together were entered in square binary matrices. A total of 36 scans were obtained during each 6 h of observation, so that 36 matrices of data for pigs lying together were built for each pen at both 6 and 8 weeks of age.

### 2.4. Construction of Matrices for Social Network Analysis

By adding up the 36 matrices of pigs’ lying together, a matrix of frequency was generated for each pen. The frequency matrix is the sum of the 36 matrices, which summarized the total number of pigs lying together over the 6 h observation period for each week. Using the lying frequency data, the half-weight association index (HWI) [22] was calculated with the equation: HWI = *x*/(*n_a_* + *n_b_*)/2; where *x* is the number of times pig *a* and pig *b* are lying together during 6 h of the observation period; *n_a_* is the number of times pig *a* is lying during the same period; and *n_b_* is the number of times pig *b* is lying during the same period. Then, a square matrix of HWI was constructed for each pen during each observation period. The column and the row of the matrix were individual pigs, and the cells were HWI between the two pigs across the column and the row. So, the matrices of HWI were weighted with values (not binary) and symmetric. As there is not an initiator or a receiver of the lying behavior, the matrices were undirected. The matrices of HWI were used for social network analysis.

### 2.5. Social Network Analysis

Social network analysis was conducted using the UCINET (version 6.6) software [24]. Each matrix of HWI for each pen during each observation period was visualized using NetDraw of UCINET.

#### 2.5.1. Social Network Measures at Pen Level

1. Density: Density describes how well individuals are connected within a network [25,26]. For binary networks, density is the sum of actual social ties divided by the number of total possible social ties. For weighted networks as in the current study, density is the sum of weights of social ties divided by the number of total possible social ties in the network [25,26]. In the current study with eight pigs per pen, the number of possible social ties were 28. So, density was calculated using the sum of a HWI matrix divided by 28. The density reflects that on average how often a pig was lying with any particular pen-mate.

2. Distribution of absent, weak, and strong social ties: ties are used to describe associations among individuals in social network analysis. Absent ties indicate that individuals do not have association or the association could be caused by chance. Individuals with weak ties are considered associated with each other, but loosely. Strong ties predict that individuals have a strong association between them. In the current study, the average HWI was 0.15 for all pigs at both 6 and 8 weeks of age. This means that on average, a pair of pigs spent 15% of their average lying time lying together. According to previous studies [27,28,29], we used 0.15, the mean value of HWI, as a cut-off value for absent ties. In other words, two pigs were considered not socially connected (absent tie) if the HWI between them was equal to or below 0.15. We estimated the distribution of absent, weak, and strong ties in each pen using the Freeman–Granovertterian Transform Procedure in UCINET. The Transform Procedure determines the break-points (cut-off values) of HWI for strong and weak ties, respectively, depending on variation in HWI for the pen. Percentage of absent, weak, and strong ties were then calculated for each pen at 6 and 8 weeks of age.

#### 2.5.2. Social Network Measures at the Individual Pig Level

1. Degree Centrality (Strength): Degree Centrality (Strength) describes direct association that an individual has with other individuals in a network. In this study, it tells how many pen mates are associated directly with each other. Degree Centrality is used for binary networks, which is the sum of actual ties that an individual has with other individuals in the network. Strength is used for weighted networks, which is the sum of tie weights. In the current study, strength is the sum of HWI that an individual pig has with all other pigs in the pen.

2. Eigenvector Centrality: Eigenvector Centrality describes how well an individual is associated with other individuals directly, as well as how well the other individuals are associated among themselves. High eigenvector is generated by a pig who is connected directly with another pig that is well connected to the remaining social group. High eigenvector centrality indicates that a specific pig is connected to a ‘social hub’—a subgroup that is well connected among themselves.

### 2.6. Statistical Analysis of the Data

For analysis of social network measures, bootstrap methods in the UCINET were used. Since HWI for each pig within a pen is not independent, social network measures were analyzed using bootstrap methods in the UCINET as much as possible. First, density of HWI matrices was tested to confirm that there were associations among pigs. Density of a HWI matrix was compared to the null hypothesis which stated that network density was zero and any deviations observed were caused by random variation. Using the procedure “Compare Network Density with Theoretical Parameter” in UCINET, we compared all matrices with the expected density = 0 using the bootstrap method with 5000 permutations. Correlation of HWI density within pen between week 6 and week 8 was calculated using the Quadratic Assignment Procedure (QAP) correlation in UCINET with 5000 permutations. This method has been recommended for analyses of correlation between matrices with the same vectors [29]. This test allows us to test whether associations among pigs in a pen at 6 weeks of age remain at 8 weeks of age. Comparison of strength and eigenvector centrality between victimized pigs and non-victimized pigs within the same pen was conducted using the paired t-test of the bootstrap method with 5000 permutations in UCINET. Effect of litter origin on social network measures was tested using the Glimmix procedure of SAS (SAS Inst. Inc., Cary, NC, USA). Data for body weight, tail score, and time budget for lying posture were also analyzed using the Glimmix procedure of SAS. To examine the variation of initial body weight between pigs assigned to different littermate treatment group, the Levene’s Test was conducted for homogeneity. To evaluate whether social association is related to tail-biting, additional analysis was conducted to compare social network measures between pens with tail-biting (with victimized pigs) and without tail-biting (without victimized pigs) using the Glimmix procedure. The models included treatment (litter origin or tail-biting incidence) as the fixed effect and pen as the experimental unit. Within the Glimmix procedure, the Poisson regression model was used for analysis of count data (tail score, and the number of absent, weak and strong ties) and the Gaussian model was used for analysis of continuous data (lying time budget, density, strength, and eigenvector centrality). Differences among means were tested by the Adjusted Tukey test, and significant differences were identified at *p* < 0.05 and trends at *p* < 0.10.

## 3. Results

### 3.1. Tail Scores and Growth Performance of Nursery Pigs

All 144 pigs remained with their pen-mates during the data collection period in the nursery barn. Pigs that were assigned to the littermate and the non-littermate treatments had lower initial weight at weaning compared to pigs assigned to the half-group of littermate treatment (Table 1). No differences (*p* = 0.76) in Standard Deviation (SD) of initial weight among treatment groups were detected by the Levene’s Test. To exclude the impact of initial body weight, weaning weight was used as a covariate in data analysis of growth performance. Litter origin did not affect body weight, average daily gain (ADG), average daily feed intake (ADFI), or gain efficiency (Gain:Feed; all *p* > 0.18). However, littermates had higher incidence of tail-biting, with 15% of pigs having a tail score of 2, compared to pigs in other groups not having any pigs with a tail score of 2 (Chi-square = 20.2, df = 4; *p* < 0.001). No pigs with a tail score of 3 or 4 were observed in this study.

### 3.2. Social Association of Nursery Pigs Measured by HWI

On average, pigs spent 60% to 68% and 83% to 89% of their time on the lying posture at 6 and 8 weeks of age, respectively (Table 2). The bootstrapped means (not shown in Table 2) of HWI density for each pen ranged from 0.097 (SD = 0.014) to 0.213 (SD = 0.024) for 6 weeks of age, and from 0.105 (SD = 0.010) to 0.208 (SD = 0.027) for 8 weeks of age. Compared with the theoretical minimal density in the null hypothesis, the z-score ranged from 4.87 to 10.98 for week 6, and from 6.03 to 12.51 for week 8, with all *p* values < 0.001, indicated that densities of HWI for all pens were greater than zero. These results suggest that pigs within a pen might be socially connected. Using time budget for the lying posture multiplied by density, we could estimate the absolute time that a pair of pigs spent lying together. For instance, a pair of pigs in non-littermate treatment spent, on average, 43 min (68% × 17.4% × 6 h × 60 min) lying together during 6 h of the observation period at 6 weeks of age. The average cut-off values for strong ties across all pens that were calculated by the Freeman–Granovertterian Transform Procedure were 0.35 (mode 0.33, range 0.19–0.43) for 6 weeks of age, and 0.39 (mode 0.44, range 0.18–0.69) for 8 weeks of age. Consequently, the cut-off values for weak ties were between the value for strong ties and the value (0.15) for absent ties for each pen.

#### 3.2.1. Effect of Litter Origin

At 6 weeks of age, litter origin affected density of HWI (Table 2). Compared with non-littermates, littermates had lower density (*p* < 0.05), with half-group of littermates being intermediate. Consistent with density, littermates had more absent ties (*p* < 0.05) and fewer weak ties (*p* < 0.05) among themselves than non-littermates, with half-group of littermates being intermediate. There were no differences in the number of strong ties among litter origin treatments. On average, each pen had two strong ties with 4 pigs involved. At the individual pig level, littermates had lower strength (*p* < 0.001) than non-littermates and half-group of littermates, suggesting that littermates might be less socially connected directly among themselves. Litter origin did not affect eigenvector centrality.

At 8 weeks of age, littermates tended to have lower density than non-littermates (*p* = 0.07). There were no differences in the number of absent, weak, or strong ties among litter origin treatments. At the individual pig level, similar to at 6 weeks of age, littermates had lower strength than non-littermates and half-group of littermates. Litter origin did not affect eigenvector centrality, similar to observations at 6 weeks of age.

#### 3.2.2. Consistency of HWI Density between 6 and 8 Weeks of Age

The correlation coefficient of density between 6 and 8 weeks of age varied widely among pens within each treatment group (Table 3). Among these pens with meaningful correlation (*p* < 0.10), two pens had a positive coefficient (*r* = 0.286 for littermates, *p* = 0.05; and *r* = 0.296 for half-group littermates, *p* = 0.06) and one had a negative coefficient (*r* = −0.288 for non-littermates, *p* = 0.07), suggesting inconsistent correlations between 6 and 8 weeks of age across pens. The median coefficient was close to zero (0.002 to −0.058) for all treatment groups, with all *p* > 0.17. So, these results suggest that density of HWI within a pen was inconsistent between the two observation periods, regardless of litter origin.

#### 3.2.3. Differences between Pens with and without Tail-Biting

Since victimized pigs were only observed in the littermate treatment, comparison between pens with and without tail-biting was conducted using the data of littermate treatment. Among the 6 pens of littermates, three pens had victimized pigs. There was no difference in density, the number of absent, weak or strong ties during either 6 weeks of age or 8 weeks of age (all *p* > 0.34; Table 4).

#### 3.2.4. Differences between Victimized and Non-Victimized Pigs

For pens with tail-biting incidence, there were 3, 1, and 3 victimized pigs in each pen, respectively (Table 5). Differences in strength and eigenvector centrality between victimized and non-victimized pigs within the same pen at both week 6 and week 8 of age were subtle and not significant (all *p* > 0.12).

## 4. Discussion

An interesting finding of this study was that pigs in the littermate pens had higher incidence of tail-biting, and lower density and strength of HWI, compared with pigs in the non-littermate pens and the half-group of littermate pens. The coincidence of more tail-biting and lower density and strength observed in littermate pens may suggest that the development of tail-biting was associated with less social connection among pigs in these pens. One may argue that there were other differences between pigs in the littermate pens and other treatment pens that may have affected HWI density. One such difference was that pigs in littermate pens had lower body weight at the start of the study than pigs in the half-group of littermate pens, with pigs in the non-littermate pens being intermediate. However, such difference in body weight was unlikely to cause differences in HWI density or strength between pigs in littermate pens and pigs in other pens. This is because the difference in initial body weight between treatment groups was subtle (on average 0.7 to 1.2 kg), compared to body weight change between 6 and 8 weeks of age. Neither density nor strength was increased from 6 to 8 weeks of age, while body weight was estimated to increase by about 6 kg during the same period. We also examined variation of initial body weight between pigs assigned to littermate treatment and pigs assigned to other treatments using the Levene’s Test for homogeneity, and no significant differences in Standard Deviation (SD) among treatment groups were found. So the differences in density and strength between pigs in littermate pens and pigs in other treatment pens were not likely related to difference in mean initial body weight or variation in initial body weight. Another interesting observation between the littermate treatment and other treatments was time budget for the lying posture. Although statistically insignificant, littermates appeared to spend less time in the lying posture (60% of observation time for littermates vs. 67–68% for pigs in other groups) compared to non-littermates and half-group littermates. Obviously, a pair of pigs did not have a chance to lie with each other if they did not lie down. Although HWI was calculated as a proportion of the total number of lying for a pair of pigs, a pig would get a zero HWI if the pig was recorded not lying at all during the 36 scan samplings. So, lower time budget for the lying posture could be one of the reasons for low HWI density observed in littermates. On the other hand, we may argue that spending less time on the lying posture itself could be a reflection of less social connection of pigs in a pen. In addition to lying together, other behaviors might be considered in the construction of social networks in pigs in future studies.

In the current study, the average density of HWI across litter origin groups was 0.15 for both 6 weeks and 8 weeks of age. This can be interpreted to mean that a given pig in a pen may lie with any other pen-mate 15% of their lying time. The absolute time that a pig spent on lying with a pen-mate during the observation period in the current study was estimated to be 35 min (0.15 × 0.65 time budget for lying × 6 h × 60 min) and 45 min (0.15 × 0.85 time budget for lying × 6 h × 60 min) at 6 weeks of age and 8 weeks of age, respectively. These results were consistent with results of a previous study by Durrell et al. [22] who reported that on average a growing-finishing pig spent 16% of its lying time or a total of 45 min during a 6 h observation period lying with a pen-mate.

We evaluated the distribution of absent, weak, and strong ties within each pen of pigs. The reason for using average density as the cut-off value was that the average density may represent chances for a pair of pigs lying together by random [22]. In other words, a pair of pigs could, just by chance, spend 15% of their lying time with any pen-mate but these pigs might not have any real social connections. In general, the cut-off values for strong ties in the current study were similar to the cut-off values used by Durrell et al. [22] to determine social preference in pigs. In that study, Durrell et al. [22] used 0.32 to determine ‘real social connections’ between pigs, which was two times the average HWI for all pigs across two observation periods in their study. So, strong ties in the current study could be considered equivalent to the ‘real social connections’ among pigs observed in Durrell’s study. In the current study, the number of strong ties was very low (about 2 ties per pen or 7% of the total possible ties in each pen). No difference in the number of strong ties was observed among litter origin treatments. The majority of social ties were weak ties and absent ties in the current study. Across all pens over the two observation periods, the number of weak ties and absent ties were 10 and 16, representing 36% and 57% of total social ties, respectively. At 6 weeks of age, littermates had fewer weak ties and more absent ties compared to non-littermates. At 8 weeks of age, differences in the number of weak ties were diminished to insignificant, but littermates still had fewer weak ties than other pigs. So, the difference in density between littermate pens and non-littermate pens appear associated with the number of weak ties and absent ties. Given the large number of weak ties compared to strong ties, and its variation among litter origin treatments, weak ties might be an important component of social networks of pigs. Farine [27] has emphasized that animals that are socially connected by weak ties may play important roles in social networks. The role of weak ties in social network of pigs may warrant further investigation.

In the current study, we used valued matrices instead of binary matrices to construct social networks. Farine [27] recommended that whenever quantitative data are possible, valued matrices should be used for social network analysis. Using the valued matrices, we were able to test correlations of density between 6 and 8 weeks of age for each pen using the QAP procedure which considers the non-independent nature of the matrices in the analysis. Results showed that correlation coefficients varied widely and did not show any tendency of correlation within a litter origin treatment. This may indicate that a pig that had a social association with another pig at 6 weeks of age may have lost that association at 8 weeks of age. This result may suggest that pigs might not form long-term associations with their pen-mates. It would be worthwhile to note that in the current study, pigs were moved to a different pen after the first video recording at 6 weeks of age. A pair of pigs that lied together in a location where they both preferred at 6 weeks of age might not lie together when they entered a new pen at 8 weeks of age. Similar results have been reported by Durrell et al. [22] that there were very few pigs (5–10%) that maintained lying together with their preferred pen-mates after they entered a new pen two weeks later. It is not clear whether the short-term social association was formed because of the preference of location in the pen or if the association was due to true social connections. Future research may need to investigate locations in the pen where pigs are lying together, social hierarchy of the pigs, and other interactions among pigs.

The incidence of tail-biting was relatively low in the current study. This was expected because tail-biting typically does not prevail until the growing-finishing period [30,31]. In the current study, only 3 pens were observed to have victimized pigs and all of these pens were in the littermate treatment. So, comparisons between tail-biting pens and non-tail-biting pens were conducted within the littermate grouping. Due to the large variation among pens within littermate treatment, no difference in any social network measures was detected between tail-biting pens and non-tail-biting pens. Then, differences in strength and eigenvector centrality between victimized pigs and non-victimized pigs were examined. With valued matrices, we could not estimate betweenness and closeness precisely because the UCINET program converts the valued matrices into binary matrices automatically. As a result, we examined whether there were any differences in strength or eigenvector centrality between victimized pigs and non-victimized pigs within the same pen. Since there were only seven victimized pigs that were observed in three pens, we could not detect any difference in strength or eigenvector centrality between victimized and non-victimized pigs. Due to the small numbers of animals involved in this study and the limited time period for data collection, we recommend using results from this study with caution. The results may need further evaluation by using more animals for extended periods of data collection.

To the best of our knowledge, this study is the first to investigate the relationship between social networks and tail-biting in pigs. Although our results suggest that low density and strength of social connections might be related to the development of tail-biting, further research is needed to identify social positions of tail-biters, victimized pigs, and non-victimized pigs. Other behaviors in addition to lying together need to be explored for construction of social networks of pigs in future studies.

## 5. Conclusions

Results of the current study suggest that littermates had a higher incidence of tail-biting, which coincided with lower HWI density, a lower strength of social connections, more absent ties, and fewer weak ties, compared to non-littermates and half-group of littermates. Less social connection with pen-mates might predispose pigs in littermate pens to development of tail-biting. Regardless of litter origin, most pigs appeared connected by weak social ties and few pigs formed strong social ties with their pen mates. Social ties among pigs do not appear to last long because there was no correlation in social association (density) among pen-mates between 6 and 8 weeks of age. Social positions of tail-biters and victimized pigs may need further investigation.

## Figures and Tables

**Table 1:** Effect of litter origin on growth performance and tail injury scores of nursery pigs.

Item	Litter Origin			SE	*p*-Value
	Non-Littermates	Half-Group of Littermates	Littermates		
# of pens	6	6	6		
Pigs/pen	8	8	8		
Body weight, kg					
4 weeks of age (weaning)	7.3 ^e,f^	7.8 ^e^	6.6 ^f^	0.36	0.06
5 weeks of age ^1^	9.3	9.3	9.1	0.28	0.52
7 weeks of age ^1^	13.7	13.5	13.3	0.81	0.72
10 weeks of age (nursery exit) ^1^	25.4	25.1	25.9	1.13	0.62
ADG, g	464	455	459	10.9	0.75
ADFI, g	671	689	663	21.4	0.72
Gain:Feed	0.683	0.656	0.709	0.018	0.19
Maximal tail score ^2^					
Score 0	47 (97.9%)	41 (85.4%)	35 (72.9%)	20.2 ^3^	0.001
Score 1	1 (2.1%)	7 (14.6%)	6 (12.5%)		
Score 2	0 (0.0%)	0 (0.0%)	7 (14.6%)		

^1^ Weaning weight was used as a covariate. ^2^ The maximal tail lesion score for each pig over 6 weeks of the study period. Values in the table are the number of pigs with the maximal tail score for each treatment group. The values in the parentheses are percent of the total number of pigs (*n* = 48) that were assigned to each treatment group. ^3^ Chi-square value (df = 4). ^e,f^ Least square means within a row without a common superscript tend to differ (*p* < 0.10).

**Table 2:** Effect of litter origin on social network measures calculated using HWI ^1^ of pigs at 6 and 8 weeks of age.

Item	Litter Origin			SE	*p*-Value
	Non-Littermates	Half-Group of Littermates	Littermates		
# of pens	6	6	6		
Pigs/pen	8	8	8		
*Time budget for lying posture, % of observation time*					
At 6 weeks of age	68.4	66.9	59.6	4.32	0.32
At 8 weeks of age	88.5	85.5	82.9	3.78	0.59
*Social network measures*					
At 6 weeks of age					
Pen level					
Density ^2^	0.174 ^a^	0.158 ^a,b^	0.119 ^b^	0.012	0.01
Distribution of ties^3^					
Absent ties	12.0 ^a^	16.5 ^a,b^	19.8 ^b^	1.66	0.01
Weak ties	14.2 ^a^	9.3 ^a,b^	6.2 ^b^	1.25	0.002
Strong ties	1.8	2.2	2.0	0.06	0.92
Pig level					
Strength	1.219 ^a^	1.104 ^a^	0.903 ^b^	0.058	0.001
Eigenvector centrality	0.347	0.347	0.335	0.012	0.74
At 8 weeks of age					
Pen level					
Density	0.170 ^e^	0.154 ^e,f^	0.135 ^f^	0.010	0.07
Distribution of ties					
Absent ties	15.7	16.2	18.3	1.64	0.50
Weak ties	10.3	10.2	7.2	1.30	0.16
Strong ties	2.0	1.7	2.5	0.58	0.61
Pig level					
Strength	1.188 ^a^	1.075 ^a^	0.943 ^b^	0.006	0.001
Eigenvector centrality	0.355	0.344	0.346	0.012	0.76

^1^ HWI = half-weight association index. ^2^ Density = sum of HWI/28. ^3^ The number of each type (absent, weak, or strong) of ties. The number of possible ties was 28 for each pen of 8 pigs. ^a,b^ Least square means within a row without a common superscript differ (*p* < 0.05). ^e,f^ Least square means within a row without a common superscript tend to differ (*p* < 0.10).

**Table 3:** Pearson correlation coefficient of HWI1 density in nursery pigs between 6 and 8 weeks of age.

Item	Coefficient		
	Median	Maximum	Minimum
Non-littermates	−0.058	0.146	−0.288 *
Half group of littermates	0.003	0.296 *	−0.253
Littermates	0.002	0.286 **	−0.165

^1^ HWI = half-weight association index. * *p* < 0.10; ** *p* = 0.05.

**Table 4:** Social network measures using HWI ^1^ for littermate pens with or without victimized pigs in the pen.

Item	Littermate Pens		SE	*p*-Value
	With Victimized Pigs	Without Victimized Pigs		
# of pens	3	3		
Pigs/pen	8	8		
At 6 weeks of age				
Density ^2^	0.125	0.113	0.013	0.56
*Distribution of ties* ^3^				
Absent ties	19.7	20.0	2.56	0.93
Weak ties	6.3	6.0	1.45	0.88
Strong ties	2.0	2.0	0.82	1.00
At 8 weeks of age				
Density	0.131	0.139	0.016	0.74
*Distribution of ties*				
Absent ties	19.3	17.3	2.54	0.60
Weak ties	6.0	8.3	1.41	0.35
Strong ties	2.7	2.3	0.94	0.81

^1^ HWI stands for half-weight association index. ^2^ Density = sum of HWI/28. ^3^ The number of each type (absent, weak, or strong) of ties. The number of possible ties was 28 for each pen of 8 pigs.

**Table 5:** Comparison of social network measures using HWI between pigs with and without tail injuries within pens of littermates.

Item	Pen ID ^1^	No. of Victimized Pigs ^2^	Pig Category		DifferenceM1-M2	*p*-Value
			Non-Victimized Pigs (M1)	Victimized Pigs (M2)		
At 6 weeks of age						
Strength	Pen 2	3	0.924	0.742	0.189	0.66
	Pen 4	1	1.267	0.840	0.427	0.19
	Pen 6	3	0.539	0.763	−0.224	0.12
Eigenvector Centrality	Pen 2	3	0.333	0.296	0.037	0.79
	Pen 4	1	0.503	0.309	0.194	0.37
	Pen 6	3	0.307	0.397	−0.090	0.22
At 8 weeks of age						
Strength	Pen 2	3	1.022	1.141	−0.119	0.33
	Pen 4	1	1.008	0.966	0.042	0.99
	Pen 6	3	0.847	0.931	−0.084	0.34
Eigenvector Centrality	Pen 2	3	0.340	0.362	−0.023	0.73
	Pen 4	1	0.367	0.340	0.027	0.86
	Pen 6	3	0.335	0.375	−0.040	0.29

^1^ Among 6 pens of littermates, 3 pens had victimized pigs of tail-biting. No victimized pigs were observed in pens of other litter origins. ^2^ Victimized pigs were defined as pigs that were recorded maximal tail score 2 or greater during the 6 weeks of nursery period between 4 and 10 weeks of age.
